# Avoiding SARS-CoV-2 infection in healthcare workers: is behavioral change the answer?

**DOI:** 10.3389/fpubh.2023.1204878

**Published:** 2023-09-19

**Authors:** Verónica Morales-Burton, Sofía A. Lopez-Ramirez

**Affiliations:** ^1^MSC Health Economics, Policy and Management, The London School of Economics and Political Science, London, United Kingdom; ^2^Department of Pediatrics, Fundación Cardioinfantil-Instituto de Cardiología, Bogotá, Colombia; ^3^Universidad del Rosario, School of Medicine and Health Sciences, Bogotá, Colombia

**Keywords:** behavioral change, SARS-CoV-2, healthcare workers, personal protection equipment (PPE), education—active learning

## Abstract

**Background:**

The COVID-19 pandemic has become an important cause of morbimortality, and healthcare workers are at the highest risk of infection. As a result, policies and guidelines have been issued, and behavioral changes have been crucial in hospitals. Among these measures, the implementation of personal protective equipment (PPE) and its appropriate use in the workplace is key to avoiding contagion, as is understanding new measures regarding patient admission, distribution, constant education on virtual platforms, among others, and changing conduct to reduce contagion. However, behavioral change interventions in healthcare workers are challenging as contextual characteristics, attributes of the intervention, and psychological factors are involved.

**Study objectives:**

The issue under investigation is the impact of COVID-19 on frontline healthcare workers in the emergency department of the Fundación Cardioinfantil (FCI). The objective was to describe their behavioral changes by studying and monitoring SARS-CoV-2 infection and their relationship through the tracing process in 2020.

**Methods:**

We conducted a case study to identify and relate the SARS-CoV-2 infection rate within the personnel in the department and the response of healthcare workers to the implementation and adherence to the use of PPE through the analysis of the different variables that contributed to behavioral change. Data were collected by a single author and analyzed by two authors using both the individual-level logic model technique and the triangulation of information, with approval from the institutional review board.

**Discussion:**

Several interventions for behavior change were registered in the data collection process. The data obtained indicated that implementation, embedding, and integration were perceived as collective and individual behavioral processes. This was supported by evidence from healthcare interventions, such as education, incentivization, training, restriction, environmental restructuring, modeling, and enablement.

**Conclusion:**

Behavioral science should be part of public health responses, as the theoretical basis suggests that change may modify the response to avoid the transmission of infectious diseases. Therefore, individuals at the highest risk appear to adopt guidance with targeted behavior adaptation interventions. Efforts to inform, instruct, and motivate healthcare workers must be continuous, and actions at the community level must be strengthened, as it is human behavior that determines the spread and mortality of infectious diseases, where community compliance to preventive behaviors plays a crucial role.

## Introduction

The COVID-19 pandemic has rapidly spread globally, with more than 6,873,477 deaths associated in 224 countries and 760,360,956 confirmed cases by March 2023 (1). Since the pandemic was declared by the World Health Organization (WHO), frontline healthcare workers have been warned of an increased risk of infection with the SARS-CoV-2 virus. When compared with the general community, frontline healthcare workers account for 10%−20% of all COVID-19 cases, given direct contact with infected patients and exposure to droplets, with a mean basic reproduction number (R0) of 1.4–4 and a mean incubation period of 6.4 days (2, 3). As a result, multiple guidelines and recommendations from policymakers, healthcare organizations, and governments have been issued, covering different areas. Among them are administrative controls, such as resource allocation, infrastructure, infection prevention, and control policies. In terms of patient care, access to laboratory testing, appropriate triage designation, adequate staff-to-patient ratios, staff training, and environmental and engineering controls must be considered to reduce viral transmission (4).

Moreover, healthcare workers need additional protection to avoid contagion and contain transmission, as they are at the highest risk of infection. In light of this, two main strategies have been suggested by health authorities worldwide: first, prioritizing the appropriate use and proper disposal of PPE to prevent droplet exposure, and second, advocating for the establishment of a dedicated area or route in the emergency department for respiratory patients. This approach ensures that access to an area without the necessary protection is restricted, subsequently mitigating the potential for infection within the healthcare environment (5). These responses are part of behavioral transformation interventions since they are designed to change health workers' conduct regarding SARS-CoV-2-infected patients, which can be analyzed using the theoretical domain framework (TDF).

The TDF integrates multiple theories of behavior and behavioral change and was initially aimed at studying the influence of evidence-based recommendations on healthcare behaviors (6). Therefore, behavioral science is a tool that should be explored and used by policymakers, healthcare managers, clinicians, and healthcare staff to design and implement interventions in healthcare, thereby enabling the seamless incorporation of clinical evidence into everyday practice. However, the effectiveness of behavior change interventions in the healthcare domain is challenging, demanding a thorough examination to delineate their merits and shortcomings. This process helps in understanding the outcomes of the intervention, especially considering that the contextual backdrop of the action in question significantly affects its results.

The following research presents a case study in the context of the COVID-19 pandemic regarding the behavior of healthcare workers in the emergency department of the Fundación Cardioinfantil. The study aims to identify and correlate the SARS-CoV-2 infection rate among the personnel in the department and the responses of the healthcare workers to the implementation and adherence to the use of PPE. The goal is to analyze how modifications in their behavior, driven by new healthcare policies tailored to the pandemic context, can affect the risk of infection, which is an example of how public health depends on behavioral change.

## Context

### Setting and population

Fundación Cardioinfantil—La Cardio is a fourth-level, internationally accredited health center in Bogota, Colombia. According to America Economia, it is the second-best health provider in the country and fifth in Latin America (7). The inpatient capacity is 347 beds, and the emergency department (A&E) provides care to more than 69,000 patients every year with a team of professionals from diverse disciplines (physicians, nurses, pharmacists, administrators, and security, among others) who are committed to offering the best available care to patients and their families. However, healthcare workers have been identified as a high-risk population with a higher likelihood of testing positive for SARS-CoV-2 infection in comparison to the general population. Estimates suggest that up to 10%−20% of all SARS-CoV-2 infections are linked to prolonged and direct exposure to infected patients (2). Therefore, the observed population in the study is the healthcare personnel of the emergency department, who come into direct contact with the highest number of patients, rendering them highly susceptible to exposure. It was within this cohort that the repercussions of behavior change interventions were observed and analyzed.

### Measures

The observed measure is the utilization of PPE. This includes surgical gloves, face shields, goggles, gowns, aprons, face masks, and respirators. These protective measures are used to prevent infection, as transmission of the SARS-CoV-2 virus predominantly occurs through close contact and exposure to droplets. While airborne transmission remains less definitively established, these precautions aim to mitigate the risks associated with different modes of airborne transmission.

### Behavioral change

The theoretical domain framework is the consensus of multiple behavioral theories used for investigating behaviors in various settings. In the context of healthcare, it has been especially useful in conducting research, where behavior change interventions are activities designed to change specific behavioral patterns required to offer the best available practice (8, 9).

Behavioral science theories, which are evidence-based principles and models created to explain and predict behavior, present a challenge when it comes to selecting just one or a few for intervention design. Behavioral theories and constructs for behavior transformation in healthcare professionals can be divided into 12 main theoretical construct domains or approaches: knowledge, skills, social/professional role identity, beliefs about capabilities, beliefs about consequences, motivation and goals, memory-attention and decision processes, environmental context and resources, social influence, emotions, behavioral regulation, and nature of behavior. The assembling of these constructs results in the theoretical domain framework, which represents behavior theories that can be individual or collective within an organization. These are used as mediators of behavioral change to address problems in implementation and analysis, to design interventions, to theorize, to test pathways of change, and to identify process measures (10).

Behavioral theories applied to emergency response or outbreaks of infectious diseases seek to understand and influence engagement in protective health behaviors, which include the Health-Belief model, the Theory Of Planned Behavior, the Protection Motivation Theory, the Precaution Adoption Process Model, and the Social Cognitive Theory, in addition to the Theory of reasoned action (11). Complex interventions in intricate settings tend to be implemented as a collective action rather than as the result of individual behavioral processes, where context is important. Human behavior and social phenomena, in general, are determined by multiple causes. Therefore, simple causal models are insufficient, and statistical interaction between causal and contextual variables is necessary (12).

Several observations and studies on the effectiveness of behavior change interventions in healthcare have been analyzed, and strengths and limitations have been presented and compared, evaluating outcomes and a change in practice. In the last decade, professional behavior change has received particular attention, and the literature regarding this involves defining and categorizing interventions, as recommended by the methodological program of the Cochrane Effective Practice and Organization of Care (EPOC) Review Group, and attempting to understand the success or failure of the intervention (13).

Designing behavior-change strategies begins with a broad approach that results in a specific intervention component. These should target specific behavioral changes, considering the targeted context and population (9). Implementing interventions for behavior change in healthcare workers has been challenging since contextual factors (i.e., attributes of the intervention) and psychological factors (i.e., vocabulary) are involved (8). The description of the behavior and why it occurs based on the theoretical domain framework will help understand the implementation.

An explanatory framework to investigate implementation in the social context is Normalization Process Theory (NPT), which is based on the assumption that individual and collective factors are crucial for the effective implementation of behavior change interventions, making everyday practices elements of interest. It involves four social mechanisms: coherence, cognitive participation, collective action, and reflexive monitoring. Coherence refers to what we do to make sense of new practices; cognitive participation refers to what we do to engage in new practices; collective action refers to what we do to enact a new practice; and reflexive monitoring is what we do to appraise the effects of a new measure. NPT as a social theory comprises implementation, embedding, and integration (13, 14). These mechanisms create implementation processes and enable us to understand the processes through which behavior change interventions are endorsed, hence focusing on action and not on beliefs, attitudes, or intentions.

Furthermore, the interventions outlined by Michie et al. (9) encompass a range of strategies. These include the following:

- Education: aimed at augmenting knowledge.
- Persuasion: utilizing communication skills to evoke positive or negative sentiments and spur action.
- Incentivization: creating an anticipation of rewards.
- Coercion: generating an expectation of punishment or cost.
- Training: for the dissemination of essential skills.
- Restriction: employing rules to curtail opportunities to engage in the targeted behavior.
- Environmental restructuring: modifying the physical or social context.
- Modeling: offering exemplars for people to aspire to or imitate.
- Enablement: increasing resources or diminishing barriers to enhance capability or opportunity (9).

Policies assume a pivotal role in driving behavior change and interventions. They encompass several facets, which include:

- Communication and marketing: utilizing different channels such as print, electronic, telephonic, or broadcast media.
- Guidelines: creating documents that recommend or mandate specific practices.
- Fiscal: leveraging the tax system to manipulate financial costs, either reducing or increasing them.
- Regulation: instituting rules or principles governing behavior or practices.
- Legislation: crafting or amending laws.
- Environmental and social planning: designing and controlling the physical or social environment.
- Service provision: encompassing the delivery of services and introducing changes to existing provisions (9).

The implementation of evidence-based practice is fundamental to achieving effective clinical outcomes. In this context, public health depends on behavioral change (9, 15). Encouraging people to follow public health measures, strengthening healthcare systems with strict infection control procedures in hospitals to protect healthcare workers and patients, preventing hospital outbreaks, and guaranteeing supplies of PPE will decrease the number of nosocomial transmissions of the disease. Encouraging adaptive and protective behavior change in human behavior determines how rapidly COVID-19 spreads and how its mortality increases.

## Key programmatic elements

### Methods

This research was conducted under a case study methodology, aiming to evaluate behavioral change among the pediatric emergency department's healthcare workers after the promotion and implementation of personal protection elements (PPE) as a measure to avoid infection by studying and monitoring the SARS-CoV-2 infection during 2020. The authors of the following study were the healthcare managers of the pediatric emergency department, a member of the COVID-19 committee, and a medical doctor involved in a different area of the pediatrics department in the institution; moreover, neither were involved in the population to be observed. This is to analyze the results and, from the obtained analysis, sustain or develop new recommendations and institutional policies to execute in the future, not only in the emergency department setting but also transversally within the different hospital departments.

### Data collection process

The data collection process was carried out by one of the authors using a manual data extraction method created by the authors, as shown in Table 1 and approved under the data management plan, without the use of computer-assisted qualitative data analysis software. Instead, we used conventional processing tools to analyze the information (i.e., MS Word and Excel), and all the data were stored in a case study database where the compilation of the information was available to ensure the reliability of the case study. There were multiple sources, and from them, many types of evidence were taken into consideration, such as documentation from the COVID-19 committee and infectious committee minutes; institutional statistics, documents, programs, plans, processes, protocols, and archival records within the virtual campus and Communications Department; interviews performed by the Epidemiology Department; observation of the adherence to the use of PPE; and reports of the Talent and Development Area in Human Resources, the Epidemiology department, and the Occupational Health and Safety Office. These sources were selected because they align with the methods implemented during the development of the regional policies toward COVID-19, the internal recommendations on infection contention strategies, and the methods to review and analyze the results obtained and develop new strategies to reduce the risk of infection. The rationale for including these sources of evidence was to perform an in-depth study of the phenomenon in the real-world context; therefore, most of the evidence came from the organization, but it also included external sources of evidence as a strength for the construct of validity (the Ministry of Labor, the Ministry of Health and Social Protection, and the District's Health Secretary), all of which are listed and available.

**Table 1:** Source of evidence.

**Source of evidence**	**FCI**	**No**.	**District Health Secretary**	**No**.	**Ministry of Labor**	**No**.	**Ministry of Health and Social Security**	**No**.	**Other**	**No**.
Documentation	COVID committee minutes Infectious committee minutes Statistics Documents Programs Plan Process Protocols Indicators	84 14 1 1 3 1 4 4 11					Guidelines	7	Guidelines	2
Archival records	Virtual campus Communications department	26 1	Circular	2	Circular	2	Circular Resolution	1 1		
Interviews	Epidemiologic trace report	1								
Observation	Adherence % in the use of PPE	4								
Reports	Talent and development management area in human resources Epidemiology department Occupational health and safety office	1 1 1								

Source: Study database.

The search for evidence within the organization was performed through the institution's quality management system (SGC) and its virtual campus, obtaining information from the educational virtual courses and materials saved and available. Other sources included the archive of the Communications Department and reports from the Talent and Development Area in Human Resources, the Epidemiology Department, and the Occupational Health and Safety Office. Sources for evidence included documents and minutes from the organization's COVID-19 committee, which was created in March 2020 to address the pandemic and explore infection outbreaks within the hospital and their assessment to improve infection prevention strategies and healthcare safety. Specific emphasis was placed on the reports of COVID-19 cases in the A&E Department involving healthcare workers.

Guidelines, protocols, instructions, plans, and processes regarding healthcare workers' protection and risk reduction were reviewed. These were crucial sources for behavior change within the A&E environment because they allowed education of the healthcare workers, recognizing and carrying out the latest updates on the PPE to be used, allowing entrance to each area and the routes within the department, thereby constantly modifying the conduct of the workers based on their adherence to these elements. The improvement plans regarding the pandemic were also incorporated, with special emphasis on Improvement Plan No. 1102, found on the SGC. It contains a complete analysis of the outbreak of COVID-19 infection in the A&E Department, with interview reports from conversations conducted by the epidemiology team under the National Health Institute's (INS) guidelines for epidemiological field research to trace COVID-19 infections (16). Direct and indirect observations were performed to assess the adherence of healthcare workers to the provided recommendations regarding the use of PPE. These recommendations were tracked and recorded as indicators in the institution's SGE. Additionally, reports related to indicators focused on COVID-19 within the organization's workforce were also considered.

Several virtual campus courses of interest to this investigation were reviewed. For instance, courses on PPE instruction and adherence to their adequate use, in addition to those on social distancing, handwashing hygiene, waste disposal, and those that include healthcare workers' support, were also used for data extraction. Reports of assistance, course completion, and hours of educational activities within the unit of analysis were examined to verify that evidence-based recommendations were being made. Archival records from the Communication Department were also reviewed, i.e., multimedia information such as videos, screensavers, bulletins, and campaigns, among other communication strategies for behavioral education. Other sources of evidence were consulted for relevant documentation regarding the local government's response to COVID-19, such as the Ministry of Labor, the Ministry of Health and Social Security, the District Health Secretary, the National Health Institute, and guidelines from infectious disease organizations.

The sources of evidence and data extracted from the organization's SGC are listed in the case study database with a code and title. However, there is limited access to the information through the quality platform or the archive. Evidence from sources outside the organization is also listed, and documents were included in the database (Table 1). All information gathered in this study is the result processes and procedures of the organization and the departments involved. To protect the human subjects involved in this research, general reports from the unit of analysis were examined collectively rather than focusing on individual subjects. Consequently, no personal identification data were inspected or mentioned.

### Data analysis

**Figure 1:**
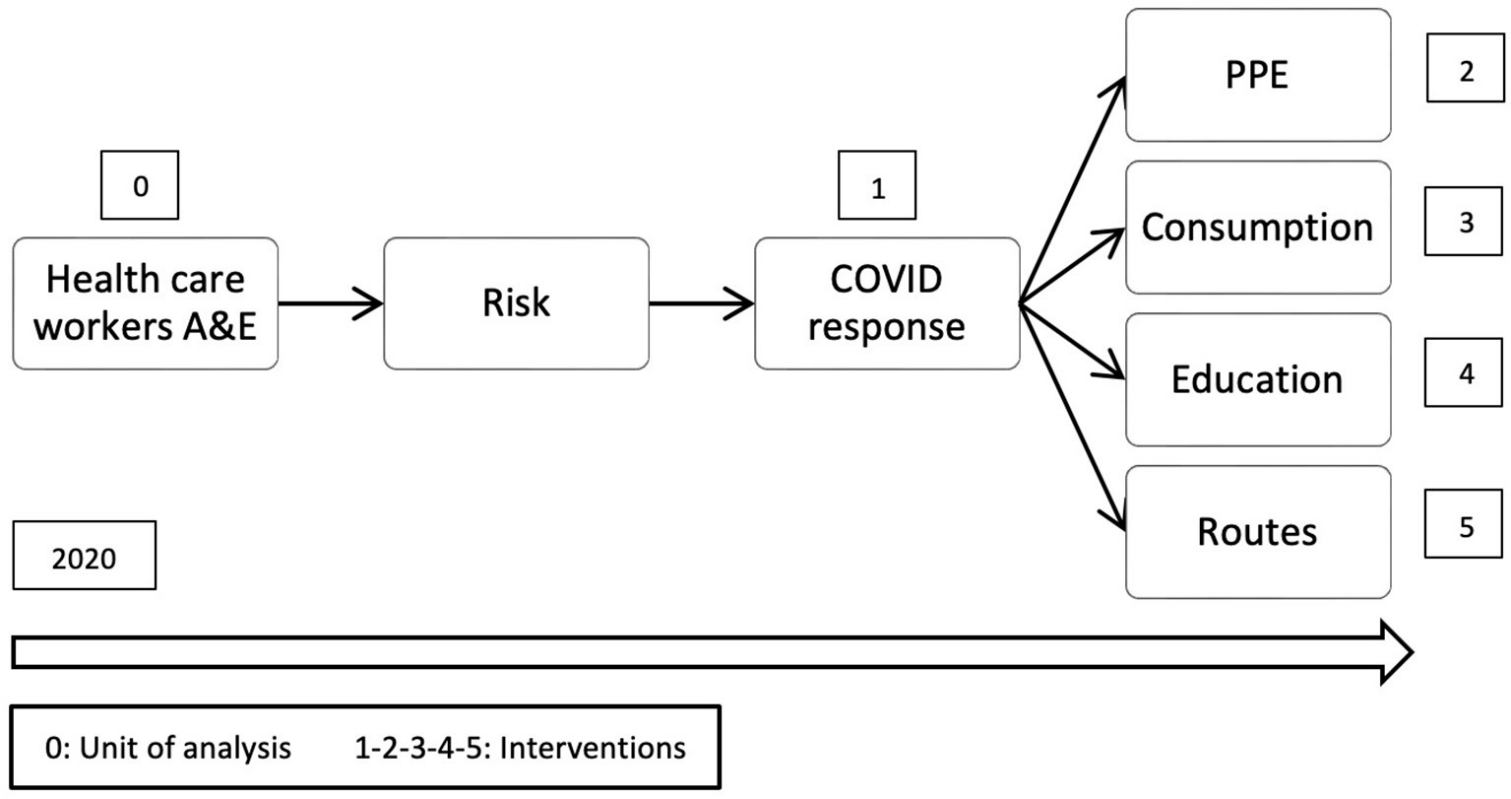
Individual-level logic model.

Using the individual-level logic model technique for analysis (Figure 1), the extracted data and evidence were coded by the author into six groups to match the findings and trace the events and interventions as follows.

#### COVID response

The interventions, understood as the actions carried out by the organization for the implementation of procedures and international and local policy due to the pandemic;

#### PPE

Evidence on actions and interventions for the implementation of recommendations for the adequate use of PPE.

#### Consumption

Evidence on PPE consumption, shortages, or issues with the supply chain in the organization.

#### Education

Evidence on training, learning, and delivering information regarding COVID-19 to people from the organization and the community.

#### Routes

Evidence on the implementation and identification of separate areas for COVID-19 patient care.

#### Risk

Evidence of increased risk of infection among healthcare workers that includes epidemiologic tracking, interview reports, and observations in the unit.

Since multiple sources of data were analyzed, triangulation was aimed at encountering converging lines of inquiry. As shown in Table 2, methodological and theoretical triangulation was applied across the sources of evidence mentioned earlier (such as documentation, institutional reports, interviews, observation, and archival records). This enabled a comprehensive exploration of study variables associated with different interventions or policies. These included educational initiatives through virtual campus courses, persuasion through communication and media, incentivization via holiday bonuses and public recognition, training in the use of PPE and proper hand hygiene, coercion through COVID safeguarding measures, restriction and restructuring with the establishment of different routes within the A&E department, exclusive admission with appropriate equipment, role modeling, and enablement focusing on attending to all A&E patients without deferrals, thus enhancing access and reducing variables. Therefore, this triangulation allowed the test validity to be based on the study variables, providing a better understanding of the situation (Table 2).

**Table 2:** Theory triangulation: intervention/policy.

**Intervention**	**Evidence**	**Policy**	**Evidence**
Education	Virtual campus courses	Communication/marketing	Campaigns COVID response
Persuasion	Communication and media	Guidelines	COVID response FCI Guidelines FCI protocols
Incentivization	Holiday bonus Public recognition	Fiscal	COVID response
Training	PPE Hand hygiene	Regulation	Social distancing Surgical mask use
Coercion	Detention strategy COVID guard	Legislation	Healthcare workers
Restriction	Routes in the A&E Department, no circulation without PPE Official communications	Environmental/social planning	Quarantines Shifts •Areas
Restructuring	Separate pediatric and adult routes in the A&E Separating areas No patient companions Implementation of telephone information	Service provision	Support services
Modeling	Leaders of the adult, pediatric, and ICU units participating as role models of the PPE in multimedia material	
Enablement	Giving attention to all patients coming into A&E, no deferrals guaranteeing access, and reducing barriers	

## Discussion

Theoretical models for behavior change in healthcare, both at the individual and community levels, are numerous; interventions, their implementation, embedding, and integration are challenging for any organization. However, efforts to implement, embed, and incorporate actions to avoid COVID-19 in the workforce at the A&E department at FCI suggest promising results. The analyzed evidence regarding the relative effectiveness of some of the interventions highlights a positive impact, as reflected in outcome measures. However, due to the intricate nature of the multi-component strategy, isolating the specific impact of individual interventions becomes challenging. The triangulation of the data extracted from the established sources and the analyzed behavioral theories lends support to the conclusion that the organization, in its response to the COVID-19 pandemic, executed a well-structured multi-component strategy plan. This plan followed the recommendations provided by the authorities and drew from experiences with previous infectious disease outbreaks. Particular emphasis was placed on protecting frontline healthcare workers, as highlighted by the implementation of PPE and strict adherence to recommended practices.

The unit of analysis of this study is the healthcare workers at the Emergency Department of the FCI with confirmed COVID-19 infections. The data extracted from the year 2020 (taking into consideration the first COVID-19 case in Colombia reported in March 2020) demonstrates congruence with institutional evidence regarding the ratio of personnel infected with SARS-CoV-2. The reports indicate that, by December 2020, 18.8% of the total workforce had contracted COVID-19 as an occupational disease. Among those testing positive for SARS-CoV-2 in the institution, 16.4% were from the A&E Department, where behavioral change efforts and specific training were implemented the earliest regarding the higher risk of infection (Table 3). Moreover, reports from the Talent and Management Area in Human Resources display the efforts of the organization in the early stages of the pandemic in Colombia, through training and supporting academic activities for the workforce, particularly those on the frontlines of care (example: A&E Department), as shown in Table 4. This was because we identified these workers with the highest probability of infection due to their exposure to droplets and close contact with respiratory patients despite their admission to inpatient care or outpatient follow-up.

**Table 3:** Positive SARS-CoV-2 report.

**2020**	**FCI**	**A&E**	**Literature**
Occupational disease	18.8%	89%	10%−20%
Community acquired	3.4%	11%	N/A
Positive SARS COV2	22.2%	16.4%	10%−20%
TOTAL	712	117	
Workforce (number)	3,200	

Source: Occupational Health and Safety Office (Study Database).

**Table 4:** Training and education.

**Courses implemented**	**Number of persons trained in the A&E department**	**Hours of training delivered**	**Type of training**	**Audience**
26	2,815	4,056	In-person Virtual	Nurses Medical staff Students Administrative staff Patients

Source: Occupational Health and Safety Office (Study Database), reports from the Talent and Development Management Area (Study Data Base).

The analyzed data suggest a proven causal effect attributed to the proper implementation, utilization, and consistent adherence to PPE by healthcare workers to avoid healthcare-acquired infections using guidelines, institutional policies, and protocols. This implementation aligns with established guidelines, institutional policies, and protocols toward averting healthcare-acquired infections. Interviews carried out by the Epidemiology Department corroborate this notion. It was revealed that frontline healthcare workers, particularly those in the Emergency Department at Fundación Cardioinfantil, were equipped with necessary PPE, comprehensive training, and essential information to effectively address infections. Notably, the organization has indicators in place that track behavioral change interventions and initiatives. These encompass the correct utilization of PPE with a systematic embedding of the procedure. Furthermore, indicators showcase the percentage of adherence to recommendations in the Emergency Department, which exhibited a significant increase to 93% by December 2020.

However, the interviews conducted by the epidemiology department to trace the source of infection, following the parameters from the government according to the INS, exposed an illogical series of events. They revealed that the community-acquired infections, which accounted for 3.4% of the total workforce with confirmed SARS-COV-2 infections, were not due to providing care to patients in COVID-19 areas or inadequate use of PPE, but were acquired at the community level, which is consistent with the data from the Occupational Health and Safety Office. It is a spurious finding from the logic model applied and rationalization of why personnel in the Emergency Department of Fundación Cardioinfantil became infected with SARS-CoV-2. Therefore, it shows that there are variables implicated in reducing the risk of infection, among them the use and adherence to PPE in the inpatient setting, with a higher rate of infection outside of the organization, probably related to avoiding the use of these elements at home or in social scenarios, which were not included and measured due to the limitations of the study.

Evidence suggests that the implementation of behavioral change interventions, such as training and education, has increased the knowledge of managing the disease in terms of the behavior of the A&E personnel and is currently part of daily practice within the institution, showing a positive response with continuously measured and evaluated outcomes. Compliance with the recommendations and policies on infection control precautions, such as the use of masks, even during off-work activities, and compliance with social distancing when indicated, division between respiratory and non-respiratory routes, and review of updated guidelines and protocols when treating positive patients, are part of the outcomes that, if reinforced, could improve the incidence of COVID-19.

In summary, the data analyzed through the process of triangulation provided evidence of behavior change interventions in healthcare (Table 2). These interventions include various aspects. First, with regard to education, there was enrollment in and assessment of different courses. Moreover, the aspect of persuasion entails the utilization of communication tools that prompt actions to prevent infection among healthcare workers within the organization. Evidence of incentivization was also found that included reward bonuses (i.e., extra holiday time) granted at the end of the year to those on the frontlines of patient care. The presence of training initiatives was also observed, involving the imparting of skills to effectively address the pandemic, showcasing the organization's solid training program, and the adoption of guidelines.

In terms of restriction, the implementation of rules aimed at diminishing opportunities for engaging in improper behaviors that escalate the risk of infection is of paramount importance. Notably, there were evident instances of environmental restructuring, underscored by recommendations tailored to managing infectious diseases. For instance, this involved segregating the pediatric emergency section from the primary adult A&E area and establishing distinct pathways for both respiratory and non-respiratory patients.

The concept of modeling is also at play, involving the provision of examples for individuals to aspire to or emulate. In this context, both managers of the organization and leaders within the emergency department played integral roles within the communication and training strategy as role models. Lastly, enablement is pursued through the execution of actions aimed at dismantling barriers for healthcare workers. This encompasses facilitating access to preferential care if symptoms manifest (Table 2).

Potential limitations should be considered when interpreting the results of this study. A case study methodology can be controversial as a research method where multiple case studies are preferred. Nevertheless, single case studies such as the one proposed are recognized as a tool in social science and have proven useful in finding explanations for behavior change where the researcher goes beyond quantitative methods to provide an in-depth explanation of behavior. Since this is an embedded study, although it refers to a single center, it includes information from the healthcare workers in the Emergency Department and considers them as a whole. The validity of the process increases with the triangulation of the extracted data. The next step to corroborate the findings would be to conduct a multi-case study involving different units of the organization (ICU, inpatient wards, and operating rooms). Additionally, another limitation of the study is the lack of access to data from similar institutions in the region to establish an infection rate comparison based on behavioral change strategies, due to confidentiality matters and the absence of public data reports. However, it would be interesting to speculate which strategies were reinforced during the same period of time in each hospital and how they impacted the risk of infection among healthcare workers in different Emergency Departments in the region.

While the case study research methodology makes it difficult to reach a generalized conclusion because of the limited sample of cases, it enables the examination of micro-level data and reveals real-life situations, providing data on the detailed behaviors of the subjects of interest, such as the ones presented in this research. Therefore, this study is an initial attempt to determine why healthcare workers on the frontlines of patient care in the Emergency Department at FCI become infected with SARS-CoV-2, despite their high adherence to recommendations concerning PPE and how a change in behavior and the application of behavioral, theories link to different outcomes.

It is important to understand the context in which the present situation is being analyzed, as FCI is the second-best hospital in the country, boasting resources and budgets that surpass those of other establishments in the region. Notably, financial constraints often hinder the execution of behavior change actions, such as training, education, and environmental planning. Therefore, interventions can be easily applied at FCI due to the availability of technology and financial capability, guaranteeing constant education, review, and implementation of various pandemic-related actions. However, the same level of sustainability may not be achievable for hospitals within the region that grapple with limited access to funding.

## Conclusion

Globally, the infection and mortality rates associated with COVID-19 rose in 2020. As a result, various efforts have been made at the governmental level to address the pandemic, such as adopting measures to contain the spread of the disease and ameliorate its consequences on the economy and labor market in addition to having policies implemented according to the countries' needs and capacities. Colombia is not an exception, and FCI has contributed to the cause.

The contribution to morbimortality by frontline workers attending COVID-19 patients must not be ignored, corresponding to at least 20% of the total infection rate (7, 17). The risk of hospital-acquired contagion results from direct contact with patients and co-workers, inadequate PPE use, and increased work-related stress, which require attention. Therefore, several policies have been executed to protect frontline workers, the ones with the highest risk of infection, while caring for COVID-19-diagnosed patients.

Within the public health response, behavioral science plays a determinant role, as it is human conduct that determines the spread and mortality of infectious diseases, where community compliance with infection-prevention behaviors, such as social distancing, the use of masks, and vaccination, is fundamental. However, efforts to reduce the transmission of infectious diseases in this specific context of SARS-CoV-2 at the community level, and to strengthen the measures in frontline healthcare, need to be reinforced.

The study presented here highlights the significance of a multi-component strategy plan aimed at safeguarding frontline healthcare workers. This strategy primarily involved the implementation of PPE and the provision of resources and strategies to facilitate comprehensive education on their proper handling, utilization, and disposal. Moreover, education on the latest adopted measures and adaptations across various sections of the institution played a pivotal role. This comprehensive approach proved essential in curbing the incidence of COVID-19 among healthcare personnel. As the risk of exposure and infection is higher, especially in the context of the emergency department workforce, behavioral change is crucial to obtaining the expected results. This was reinforced by prompt, continuous education through institutional communications and media within the organization, lectures, and interactive courses on the institution's virtual campus based on global and local policies and recommendations based on previous infectious disease outbreaks, allowing admission to respiratory areas only to personnel who had adequate equipment and could prove their appropriate use and disposal, in addition to holiday bonuses.

This was demonstrated during the interviews and the analysis of the data, where we reviewed positive SARS-CoV-2 tests. This review highlighted a direct relationship between healthcare workers' adherence to PPE guidelines to prevent healthcare-acquired infections and the incidence of occupational SARS-CoV-2 infections. In contrast, infections acquired within the community stem from individuals lacking access to all the equipment needed to prevent the infection or choosing not to use PPE due to familiarity with their contacts. Workers need to include individual and collective variables. These are important for effectively incorporating behavioral change interventions delivered as a multi-component strategy, which is challenging to evaluate as it is difficult to isolate the effect of a particular intervention. The original contributions presented in the study are included in the article/supplementary material, and further inquiries can be directed to the corresponding author.

In summary, the theoretical base suggests acknowledging behavioral change as the answer to avoiding infectious diseases, although the implementation of actions and their effectiveness in different circumstances are difficult to measure. Individuals with the highest risk, such as frontline A&E healthcare workers, adopt recommendations within their work environment when targeted behavior change interventions are established, but do not apply them at the community level, with infection striking outside their work environment. The Fundación Cardioinfantil has followed policy recommendations, and concrete efforts have been made to inform, train, motivate, and give confidence to those on the frontlines of patient care in the A&E department. In response to the pandemic, with many patients seeking medical care, this has been evaluated as appropriate within the context, but continued efforts to promote social distancing and self-care at the community level for the organization's workforce need to be strengthened.

## Data availability statement

The original contributions presented in the study are included in the article/supplementary material, further inquiries can be directed to the corresponding authors.

## Ethics statement

The studies involving humans were approved by Fundación Cardioinfantil-La Cardio Ethics Committee and Institutional Review Board. The studies were conducted in accordance with the local legislation and institutional requirements. Written informed consent for participation was not required from the participants or the participants' legal guardians/next of kin in accordance with the national legislation and institutional requirements.

## Author contributions

VM-B contributed to the first draft of the manuscript. SL-R contributed to the context and to the editing and review of the manuscript. All authors contributed to the conceptualization of this viewpoint, made substantial contributions to the design of the work, contributed to the bibliography and the refinement of the final version, and have approved and accepted responsibility for the entire content of the final manuscript.
